# Chronic synaptic insulin resistance after traumatic brain injury abolishes insulin protection from amyloid beta and tau oligomer-induced synaptic dysfunction

**DOI:** 10.1038/s41598-019-44635-z

**Published:** 2019-06-03

**Authors:** Whitney Franklin, Balaji Krishnan, Giulio Taglialatela

**Affiliations:** 1 Mitchell Center for Neurodegenerative Diseases, Department of Neurology, University of Texas Medical Branch, Galveston, Texas 77555 USA; 2 Department of Neuroscience, Cell Biology & Anatomy, University of Texas Medical Branch, Galveston, Texas 77555 USA

**Keywords:** Alzheimer's disease, Alzheimer's disease, Risk factors, Brain injuries, Cellular neuroscience

## Abstract

Traumatic brain injury (TBI) is a risk factor for Alzheimer’s disease (AD), although the mechanisms contributing to this increased risk are unknown. Insulin resistance is an additional risk factor for AD whereby decreased insulin signaling increases synaptic sensitivity to amyloid beta (Aβ) and tau. Considering this, we used rats that underwent a lateral fluid percussion injury at acute and chronic time-points to investigate whether decreased insulin responsiveness in TBI animals is playing a role in synaptic vulnerability to AD pathology. We detected acute and chronic decreases in insulin responsiveness in isolated hippocampal synaptosomes after TBI. In addition to assessing both Aβ and tau binding on synaptosomes, we performed electrophysiology to assess the dysfunctional impact of Aβ and tau oligomers as well as the protective effect of insulin. While we saw no difference in binding or degree of LTP inhibition by either Aβ or tau oligomers between sham and TBI animals, we found that insulin treatment was able to block oligomer-induced LTP inhibition in sham but not in TBI animals. Since insulin treatment has been discussed as a therapy for AD, this gives valuable insight into therapeutic implications of treating AD patients based on one’s history of associated risk factors.

## Introduction

Alzheimer’s disease (AD) is a devastating neurodegenerative disorder for which there is no resolving therapeutic intervention. While the initial cause of this disease is still unknown, there are many innate and event-triggered factors that increase the risk of developing AD. For example, type 2 diabetes (T2DM)^1–3^, central insulin resistance^4,5^, obesity^6^, traumatic brain injury (TBI)^7–9^, mitochondrial dysfunction^10,11^, and neuroinflammation^12,13^ are all known risk factors. In general, it is believed that synaptic dysfunction underlies the initiation and progression of the disease^14,15^. It has become increasingly evident that oligomeric aggregates of both amyloid beta (Aβ) and tau contribute to this synaptic dysfunction that precedes the cognitive decline seen in AD^16,17^. Notably, decreased insulin receptor function increases synaptic sensitivity to the binding of and dysfunction caused by Aβ^18^, and insulin and insulin-sensitizing therapy has been shown to be effective for cognition in mouse models of AD as well as in patients with mild cognitive impairment (MCI) or early AD^18–22^. Furthermore, AD patients exhibit insulin resistance and decreased insulin signaling response in the hippocampus^23,24^. This multitude of evidence strongly indicates the existence of an intimate relationship between synaptic insulin responsiveness and neuronal sensitivity to AD neuropathology. Several groups have reported hyperglycemia after TBI and found that uncontrolled blood glucose levels lead to a poorer outcome and recovery^25,26^. Previous reports have also found an increased mortality after head injury in people with T2DM^27^. While one group has reported acute decreased insulin signaling in the CNS after TBI^28^, no studies have investigated TBI-driven insulin resistance at the synapses, particularly in relation to synaptic vulnerability to Aβ and tau. Therefore, in the present work, we investigated changes in synaptic insulin responsiveness in the hippocampus after TBI in relation to an increased risk to AD pathology-driven synaptic dysfunction and protection by insulin treatment. Overall, our results indicate that, after TBI, hippocampal synapses become chronically insulin resistant and, contrary to sham animals, unresponsive to the beneficial effect of insulin therapy as a treatment against synaptic impairments due to Aβ and tau oligomers. Thus, this work further illustrates the importance of considering a prior history of associated risk factors and how these may impact the efficacy of particular treatments that are being investigated for AD in the general population.

## Results

### Synaptosomal insulin responsiveness is chronically decreased after TBI

**Figure 1:**
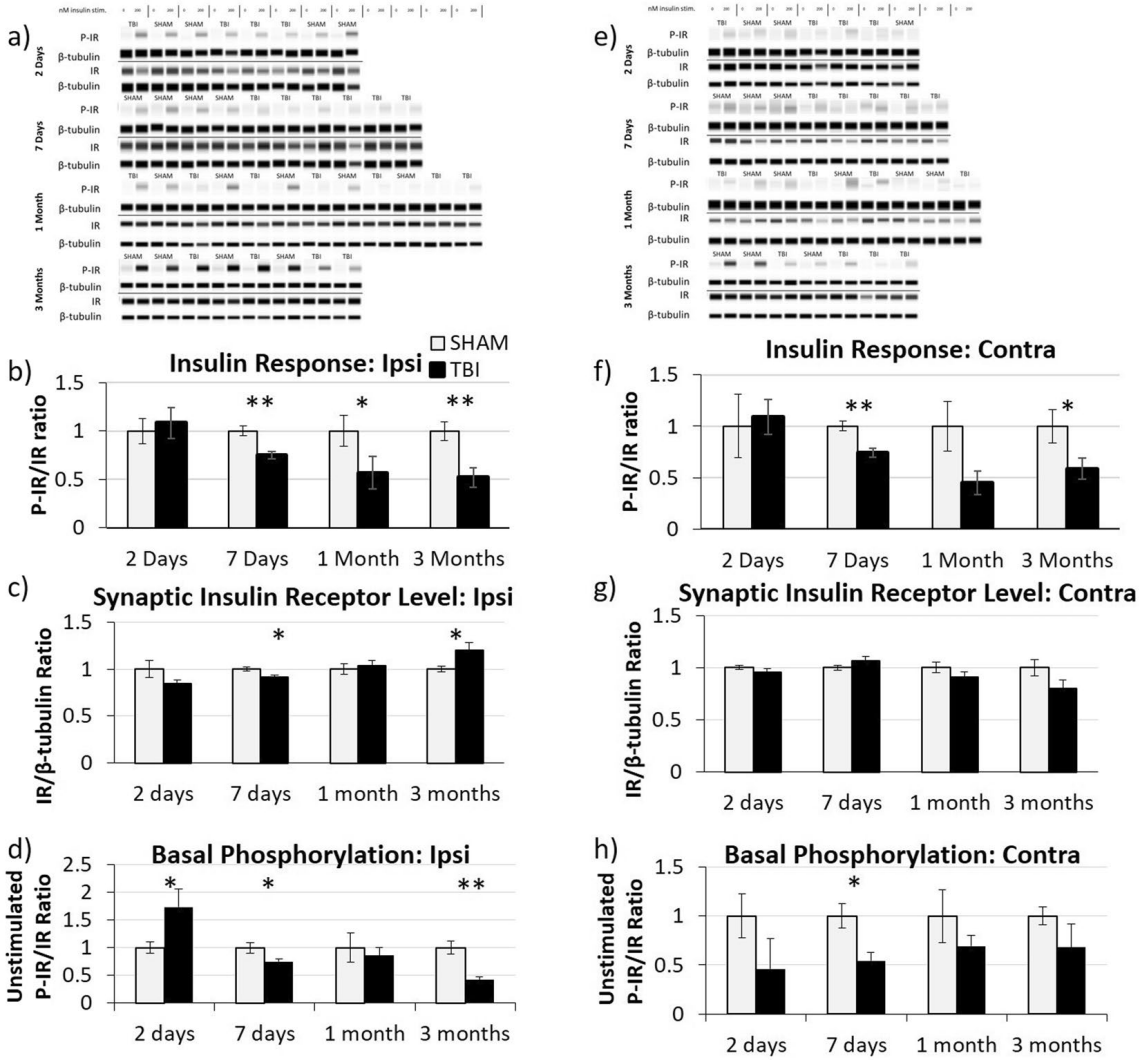
Acute and chronic decreases in synaptic insulin responsiveness. *Ex vivo* insulin responsiveness assays were conducted on isolated hippocampal synaptosomes at various time-points after traumatic brain injury. Representative Wes analysis of insulin-stimulated and unstimulated synaptosomes isolated from the ipsilateral hippocampus **(a)** at 2 DPI (n = 4 for both sham and TBI), 7 DPI (n = 4 sham, n = 6 TBI), 1 MPI (n = 5 sham, n = 7 TBI), and 3 MPI (n = 4 for both sham and TBI) animals. Quantitative graph of Wes analysis showing **(b)** the ratio of insulin-stimulated P-IR/β-tubulin to IR/β-tubulin demonstrating that synaptosomal insulin responsiveness is chronically decreased after TBI, **(c**) IR/β-tubulin showing the changes in insulin receptor level at the synapse, and **(d)** the ratio of unstimulated P-IR/β-tubulin to IR/β-tubulin as an indication of basal phosphorylation levels of the receptors. Representative Wes analysis of insulin-stimulated and unstimulated synaptosomes isolated from the contralateral hippocampus **(e**) from 2 DPI (n = 3 sham, n = 4 TBI), 7 DPI (n = 3 sham, n = 5 TBI), 1 MPI (n = 5 sham, n = 4 TBI), and 3 MPI (n = 3 sham, n = 4 TBI) animals with corresponding quantitative graphs of **(f**) the ratio of insulin-stimulated P-IR/β-tubulin to IR/β-tubulin, **(g**) IR/β-tubulin, and **(h)** the ratio of unstimulated P-IR/β-tubulin to IR/β-tubulin. A Wes assay was run with samples probed for P-IR and β-tubulin as a loading control. Samples were then run through a second Wes assay and probed for total IR and β-tubulin. Full length Wes images are presented in Supplementary Figs S1 and S2. Statistical significance was determined by unpaired t-test analysis. Error bars represent standard error. *p < 0.05; **p < 0.01.

We first longitudinally characterized synaptic insulin responsiveness in the hippocampus of rats at different time points after fluid percussion injury (FPI) using the *ex vivo* insulin stimulation method described in the Materials and Methods section. Briefly, we isolated synaptosomes from sham and TBI animals at different times after injury (2 days post-injury (DPI), 7 DPI, 1 month post-injury (MPI), and 3 MPI), exposed them to insulin in the presence of ATP, and analyzed their extent of insulin receptor (IR) phosphorylation (i.e. activation) via Wes for both the ipsilateral (Fig. 1a–d) and contralateral hippocampi (Fig. 1e–h).

In the ipsilateral hippocampus, we found a significant decrease in synaptic insulin responsiveness by 7 DPI (p = 0.0041) (Fig. 1b) that was further decreased by 3 MPI (p = 0.0067). Moreover, we observed a significantly decreased level of IR at the synapse at 7 DPI (p = 0.0115), as compared to sham-injured animals (Fig. 1c). However, this decrease in IR level cannot account for the decreased insulin response at this time-point after injury as the ratio of the phosphorylation of the insulin receptor normalized to the total insulin receptor is still significantly decreased at this time point. At 1 MPI, the synaptic IR level was normalized back to the level of sham-injured animals, and there was a further significant increase in IR level at the synapse at 3 MPI (p = 0.0396) which could be indicative of an attempted compensatory mechanism. We also found a significant increase in basal (unstimulated) level of IR phosphorylation at 2 DPI (p = 0.0357) (Fig. 1d), whereas at both 7 DPI and 3 MPI, the basal level of IR phosphorylation was significantly decreased (for 7DPI p = 0.0359) (for 3MPI p = 0.0049).

In the contralateral hippocampus, there was a significant decrease in IR response to insulin at 7 DPI (p = 0.0050) and 3 MPI (p = 0.0378) (Fig. 1f). Interestingly though, unlike the ipsilateral hippocampus, we found no difference in synaptic IR level in the contralateral hippocampus at any of the time points studied (Fig. 1g). There was, however, a significant decrease in the basal level of IR phosphorylation in TBI animals at 7 DPI (p = 0.0185) that returned to normal levels by 1 MPI (Fig. 1h).

In summary, we found a significant decrease in the synaptic insulin receptor’s response to insulin at 7 DPI, 1 MPI, and 3 MPI but not at 2 DPI. These data indicate that there are chronic deficits in synaptic insulin responsiveness in both the ipsilateral and contralateral hippocampi after lateral FPI.

### *Ex vivo* Aβ-oligomer synaptic binding is not affected after TBI

**Figure 2:**
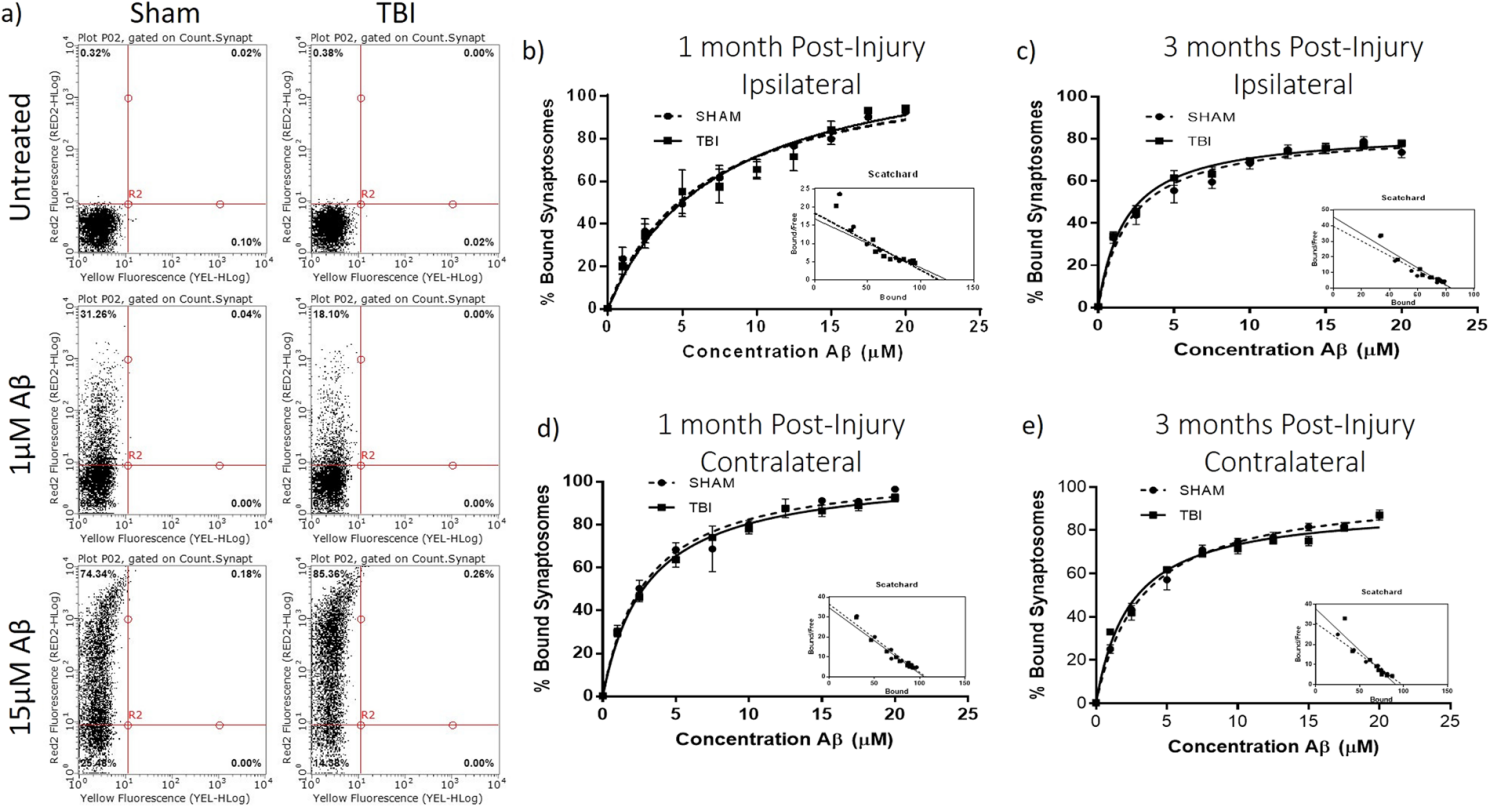
Flow cytometry analysis of *ex vivo* amyloid-beta oligomer binding curves. (**a)** Representative flow cytometry analysis of pooled synaptosomes isolated from 1-month post-injury sham and TBI animals challenged with increasing concentrations of Aβ oligomers tagged with HyLite Fluor 647. Michaelis-Menton graphs from three separate binding curve analyses showing the percent of synaptosomes with bound Aβ oligomers determined by flow cytometry analysis for synaptosomes isolated from the ipsilateral hippocampus at **(b)** 1-month post-injury (n = 5 sham, n = 7 TBI) and **(c)** 3 months post-injury (n = 4 for both sham and TBI)and from the contralateral hippocampus at **(d)** 1-month post-injury (n = 5 sham, n = 7 TBI) and (**e)** 3 months post-injury (n = 4 sham, n = 3 TBI) with Scatchard plot transformation. Error bars represent standard error.

To investigate whether the decreased synaptic insulin responsiveness observed after FPI may lead to increased synaptic sensitivity to Aβ binding, we studied *ex vivo* Aβ oligomer binding on isolated synaptosomes from sham and TBI animals at 1 month and 3 months after injury and evaluated the maximum binding capacity (B_max_) and affinity (K_d_) of Aβ binding with Scatchard plot analysis. We did not find any differences in Aβ binding at synapses in the ipsilateral hippocampi at either the 1 MPI (Fig. 2b) (sham B_max_ = 117.2 +/− 7.448 and K_d_ = 6.392 +/− 1.112 μM; TBI B_max_ = 125.1 +/− 11.39 and K_d_ = 7.496 +/− 1.734 μM) or 3 MPI (Fig. 2c) (sham B_max_ = 83.74 +/− 2.753 and K_d_ = 2.112 +/− 0.322 μM; TBI B_max_ = 83.76 +/− 1.795 and K_d_ = 1.836 +/− 0.195 μM) time points. We also did not find differences in Aβ binding at synapses in the contralateral hippocampi at the 1 MPI (sham B_max_ = 106.6 +/− 3.774 and K_d_ = 2.925 +/− 0.412 μM; TBI B_max_ = 104.8 +/− 2.439 and K_d_ = 3.028 +/− 0.276 μM) time point (Fig. 2d). However, we did find a significantly decreased Aβ binding after TBI in contralateral hippocampal synaptosomes at 3 MPI when considering both B_max_ and K_d_ (sham B_max_ = 98.45 +/− 2.239 and K_d_ = 3.205 +/− 0.278 μM; TBI B_max_ = 91.14 +/− 2.178 and K_d_ = 2.414 +/− 0.251 μM) (for B_max_ p = 0.0228) (for K_d_ p = 0.0390) (Fig. 2e). These results suggest that despite onset of insulin resistance, synapses are not more susceptible to Aβ oligomer binding at the chronic time points of 1 month or 3 months after TBI.

### *Ex vivo* tau-oligomer synaptic binding is not affected after TBI

**Figure 3:**
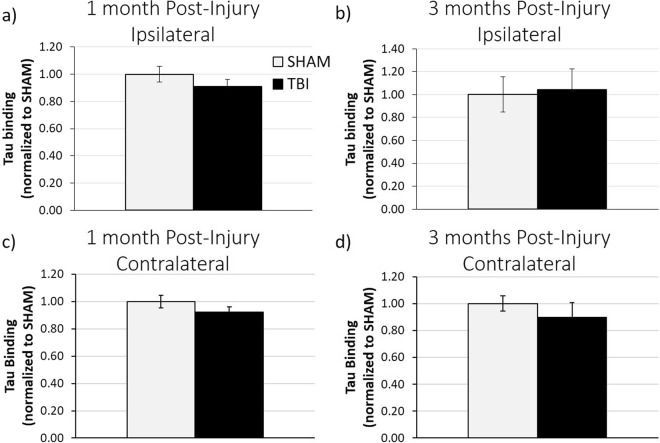
ELISA analysis of *ex vivo* tau oligomer binding assays. Quantification of 2 µM tau-oligomer *ex vivo* binding on isolated synaptosomes determined by tau-5 ELISA analysis in the ipsilateral hippocampus at **(a)** 1 month post-injury (n = 5 sham, n = 7 TBI) and **(b)** 3 months post-injury (n = 4 for both sham and TBI) and in the contralateral hippocampus at **(c)** 1 month post-injury (n = 5 sham, n = 7 TBI) and **(d)** 3 months post-injury (n = 4 sham, n = 3 TBI). Error bars represent standard error.

In order to determine whether the chronically decreased synaptic insulin responsiveness after FPI would affect synaptic vulnerability to tau oligomers, we performed *ex vivo* tau binding on hippocampal synaptosomes isolated from both sham and TBI animals at 1 month and 3 months post-injury (Fig. 3). We found that both ipsilateral and contralateral hippocampal synaptosomes from TBI animals bound similar levels of exogenously added tau oligomers as compared to sham animals at both 1 MPI and 3 MPI. These data suggest that, similar to what we found for Aβ-oligomer binding, synapses are not more susceptible to tau oligomer binding at the chronic time points of 1 month or 3 months after TBI.

### Electrophysiology assessment

**Figure 4:**
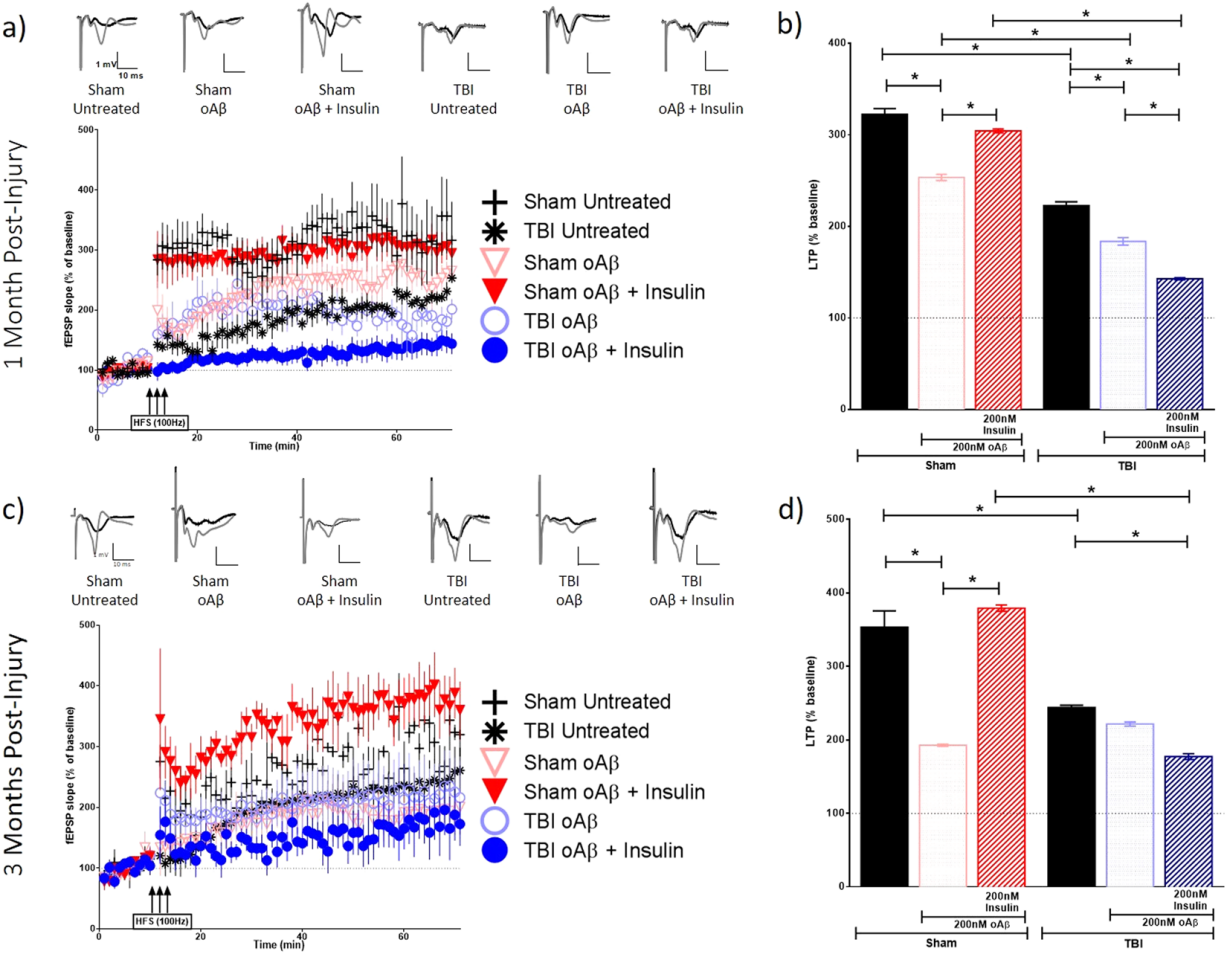
Long-term potentiation (LTP) in ipsilateral hippocampal slices exposed to Aβ oligomers in the presence or absence of insulin. Schaffer collateral field recordings were performed to determine oligomer-induced LTP impairment in slices from sham and TBI animals. Graphs of fEPSP’s slopes as a percentage of the baseline with representative traces for each condition at (**a**) 1-month post-injury and (**c**) 3 months post-injury. Graphs showing the average of the fEPSP slope for the final 10 minutes (time points 50–60 minutes post high frequency stimulation) as an indication of LTP for each condition at (**b**) 1-month post-injury and (**d**) 3 months post-injury. 1 MPI n = 4 animals and 3–6 slices per condition; 3 MPI n = 3–5 animals and 3–7 slices per condition. One-way ANOVA with Bonferroni’s post hoc analysis was used to determine statistical significance. Error bars represent standard error. *p < 0.05.

**Figure 5:**
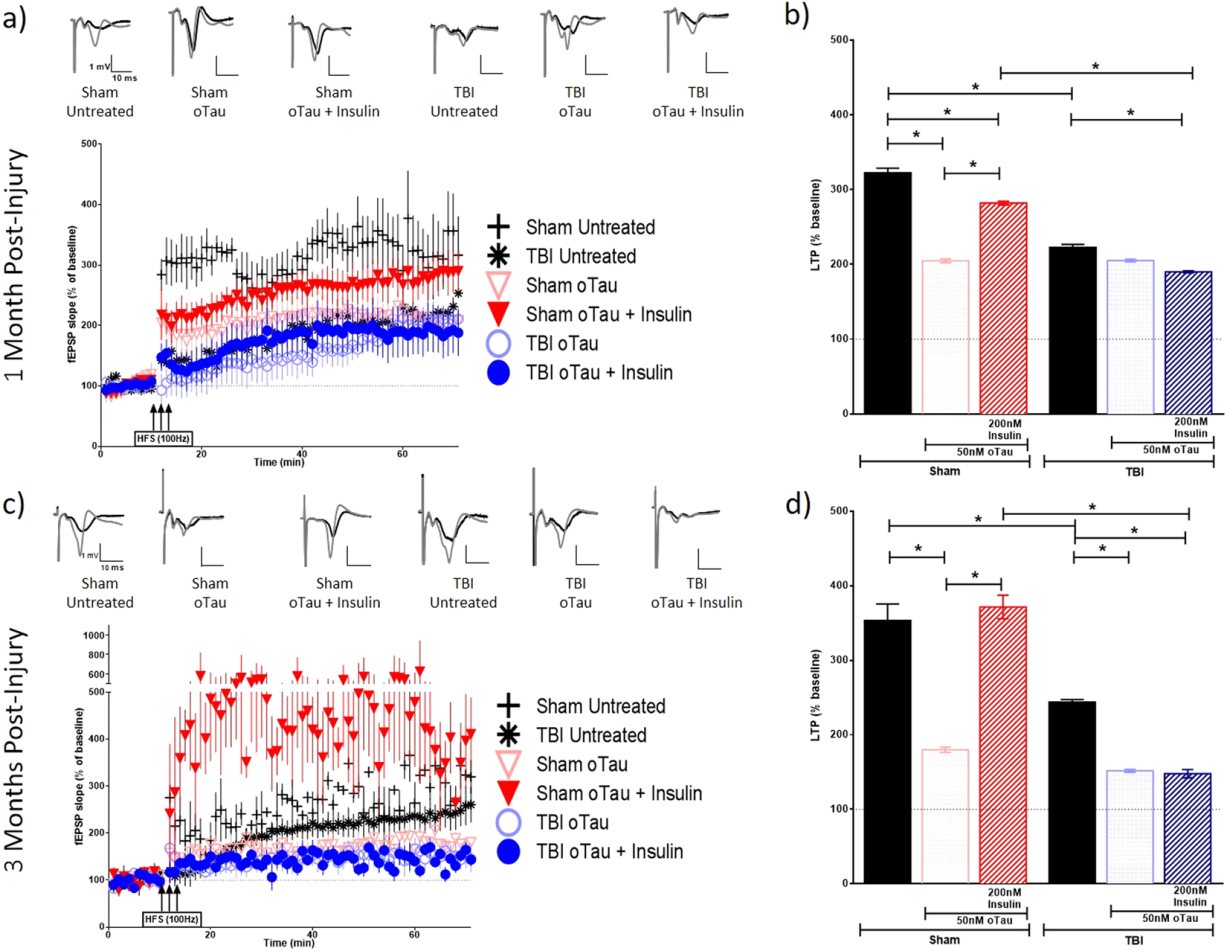
Long-term potentiation (LTP) in ipsilateral hippocampal slices exposed to tau oligomers in the presence or absence of insulin. Schaffer collateral field recordings were performed to determine oligomer-induced LTP impairment in slices from sham and TBI animals. Graphs of fEPSP’s slopes as a percentage of the baseline with representative traces for each condition at (**a**) 1 month post-injury and (**c**) 3 months post-injury. Graphs showing the average of the fEPSP slope for the final 10 minutes (time points 50–60 minutes post high frequency stimulation) as an indication of LTP for each condition at (**b**) 1 month post-injury and (**d**) 3 months post-injury. 1 MPI n = 4 animals and 3–6 slices per condition; 3 MPI n = 3–5 animals and 3–7 slices per condition. One way ANOVA with Bonferroni’s post hoc analysis was used to determine statistical significance. Error bars represent standard error. *p < 0.05.

**Figure 6:**
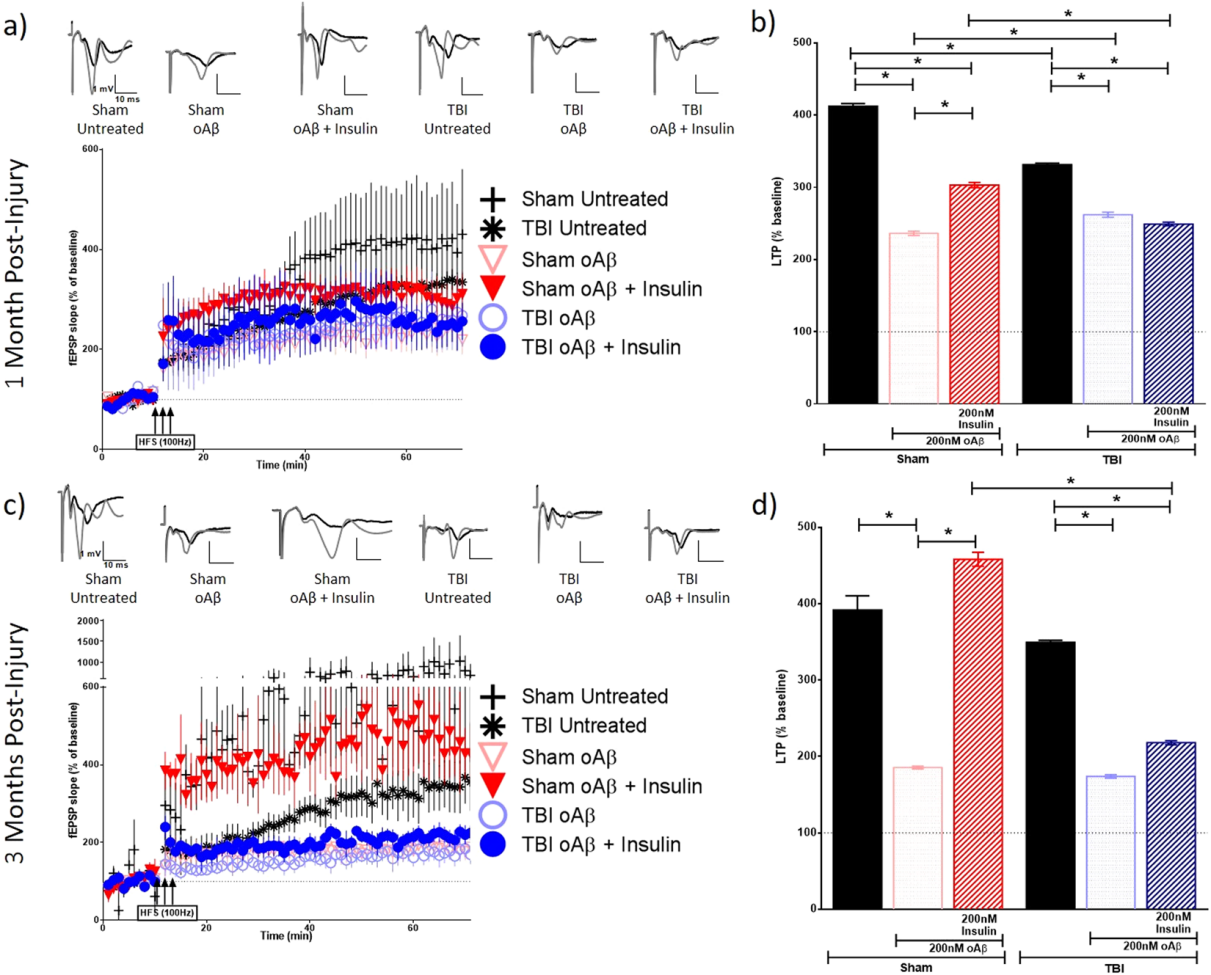
Long-term potentiation (LTP) in contralateral hippocampal slices exposed to Aβ oligomers in the presence or absence of insulin. Schaffer collateral field recordings were performed to determine oligomer-induced LTP impairment in slices from sham and TBI animals. Graphs of fEPSP’s slopes as a percentage of the baseline with representative traces for each condition at (**a**) 1-month post-injury and (**c**) 3 months post-injury. Graphs showing the average of the fEPSP slope for the final 10 minutes (time points 50–60 minutes post high frequency stimulation) as an indication of LTP for each condition at (**b**) 1-month post-injury and (**d**) 3 months post-injury. 1 MPI n = 4 animals and 3–6 slices per condition; 3 MPI n = 3–5 animals and 3–7 slices per condition. One-way ANOVA with Bonferroni’s post hoc analysis was used to determine statistical significance. Error bars represent standard error. *p < 0.05.

**Figure 7:**
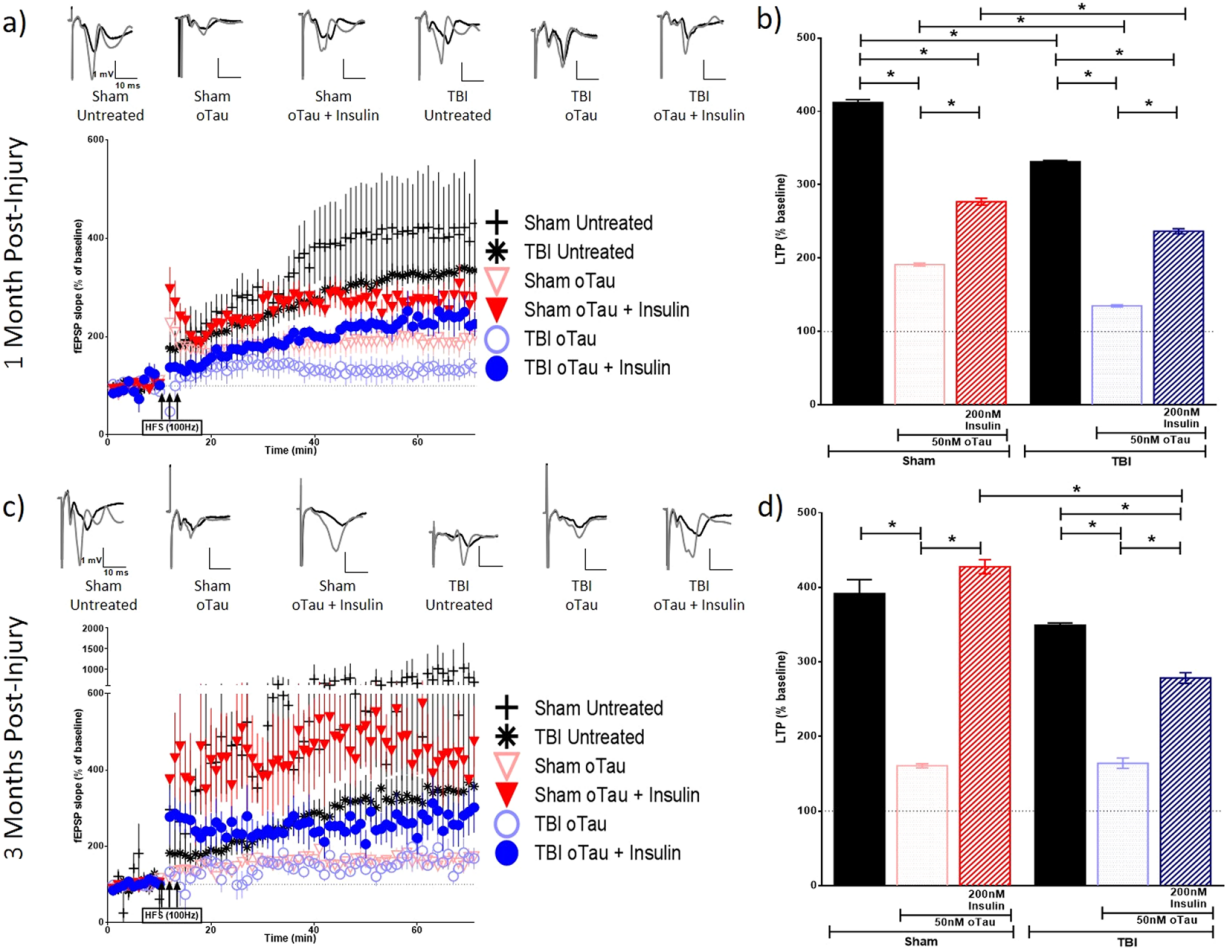
Long-term potentiation (LTP) in contralateral hippocampal slices exposed to tau oligomers in the presence or absence of insulin. Schaffer collateral field recordings were performed to determine oligomer-induced LTP impairment in slices from sham and TBI animals. Graphs of fEPSP’s slopes as a percentage of the baseline with representative traces for each condition at (**a**) 1-month post-injury and (**c**) 3 months post-injury. Graphs showing the average of the fEPSP slope for the final 10 minutes (time points 50–60 minutes post high frequency stimulation) as an indication of LTP for each condition at (**b**) 1-month post-injury and (**d**) 3 months post-injury. 1 MPI n = 4 animals and 3–6 slices per condition; 3 MPI n = 3–5 animals and 3–7 slices per condition. One-way ANOVA with Bonferroni’s post hoc analysis was used to determine statistical significance. Error bars represent standard error. *p < 0.05.

While we did not observe any significant change in hippocampal synaptic vulnerability to the binding of Aβ and tau oligomers in TBI animals, we wanted to investigate whether the impact of these oligomers on synaptic function was affected by the injury, nonetheless. We sought to additionally evaluate whether an application of insulin could block the suppression of LTP induced by Aβ and tau oligomers. Hippocampal slices of the ipsilateral (Figs 4 and 5) and contralateral (Figs 6 and 7) hemispheres prepared from sham and TBI animals were treated with 200 nM of Aβ oligomers, 50 nM of tau oligomers, and/or 200 nM insulin for 1 hour prior to recording. A one-way analysis of variance (ANOVA) was calculated for both the 1 MPI and 3 MPI time points. The Bonferroni’s post hoc test was used to determine statistical significance between the LTP of each condition. The ipsilateral and contralateral hippocampi for sham and TBI animals fail to show significant statistical difference as determined by input-output curves, suggesting that there are no appreciable differences in the basal synaptic strength (Supplementary Fig. S3 and Table S1).

At 1 MPI, we saw a reduction in the magnitude of LTP from untreated brain slices in both the ipsilateral (p < 0.0001) and contralateral (p < 0.0001) hippocampi from the injured animals vs sham animals. At 3 MPI, we found this same significant reduction in the magnitude of LTP from untreated brain slices from the injured animals vs sham animals in the ipsilateral hippocampus (p < 0.0001).

At 1 MPI, in slices treated with Aβ oligomers for 1 hour prior to recording, the magnitude of LTP was significantly lower in the TBI versus sham group in the ipsilateral hippocampus (p < 0.0001) (Fig. 4b). However, in the contralateral hippocampus, we saw the opposite effect where the magnitude of LTP, while only modestly higher, was significantly increased compared to slices from sham animals (p = 0.0021) (Fig. 6b). For tau oligomer-treated slices at 1 MPI, the magnitude of LTP was significantly decreased in slices taken from TBI versus sham animals in the contralateral hippocampus (p < 0.0001) (Fig. 7b). However, for 3 MPI, in both hemispheres’ hippocampi, we found no significant differences in LTP suppression due to either the Aβ (Figs 4d and 6d) or tau oligomer (Figs 5d and 7d) treatments for the TBI versus sham group.

The 200 nM insulin treatment during the Aβ oligomer-challenge was able to block LTP suppression in sham hippocampal slices at both time points in the ipsilateral (for both 1 MPI and 3 MPI p < 0.0001) (Fig. 4b,d) and contralateral hemispheres (for both 1 MPI and 3 MPI p < 0.0001) (Fig. 6b,d). The 200 nM insulin treatment was also able to block tau oligomer-induced LTP suppression in sham hippocampal slices at both time points in the ipsilateral (for both 1 MPI and 3 MPI p < 0.0001) (Fig. 5b,d) and contralateral hemispheres (for both 1 MPI and 3 MPI p < 0.0001) (Fig. 7b,d). Interestingly, the insulin treatment did not block the Aβ-induced LTP inhibition in slices from TBI animals at either time point in both the ipsilateral (Fig. 4bd) and contralateral (Fig. 6b,d) hippocampi. While the insulin provided no protection against the tau-induced LTP reduction in slices from TBI animals in the ipsilateral hemisphere (Fig. 5b,d), we did find that insulin provided a partial protection against tau in the contralateral hemisphere at both time points (for both 1 MPI and 3 MPI p < 0.0001) (Fig. 7b,d).

Collectively, these results suggest that TBI does not generally affect vulnerability of synapses to Aβ or tau oligomer-induced LTP impairments to a higher degree from that seen in sham animals at 1 or 3 months after injury. Insulin alone did not increase expression of LTP but rather significantly decreased LTP in both sham and TBI animal slices (Supplementary Fig. S4). However, insulin rescued the suppression of LTP induced by Aβ or tau oligomers in both the ipsilateral and contralateral hippocampus from sham animals. Importantly, though, this protective effect of insulin was not seen for either Aβ or tau in the ipsilateral hippocampus of TBI animals at either time point as well as for Aβ impairment at either time point in the contralateral hippocampus. This data further corroborates the insulin resistance in the hippocampus after TBI shown by the *ex vivo* insulin stimulation analysis previously described and provides a valuable demonstration that even a significant administration of insulin cannot overcome this phenomenon.

### SOCS3 synaptic level after TBI

**Figure 8:**
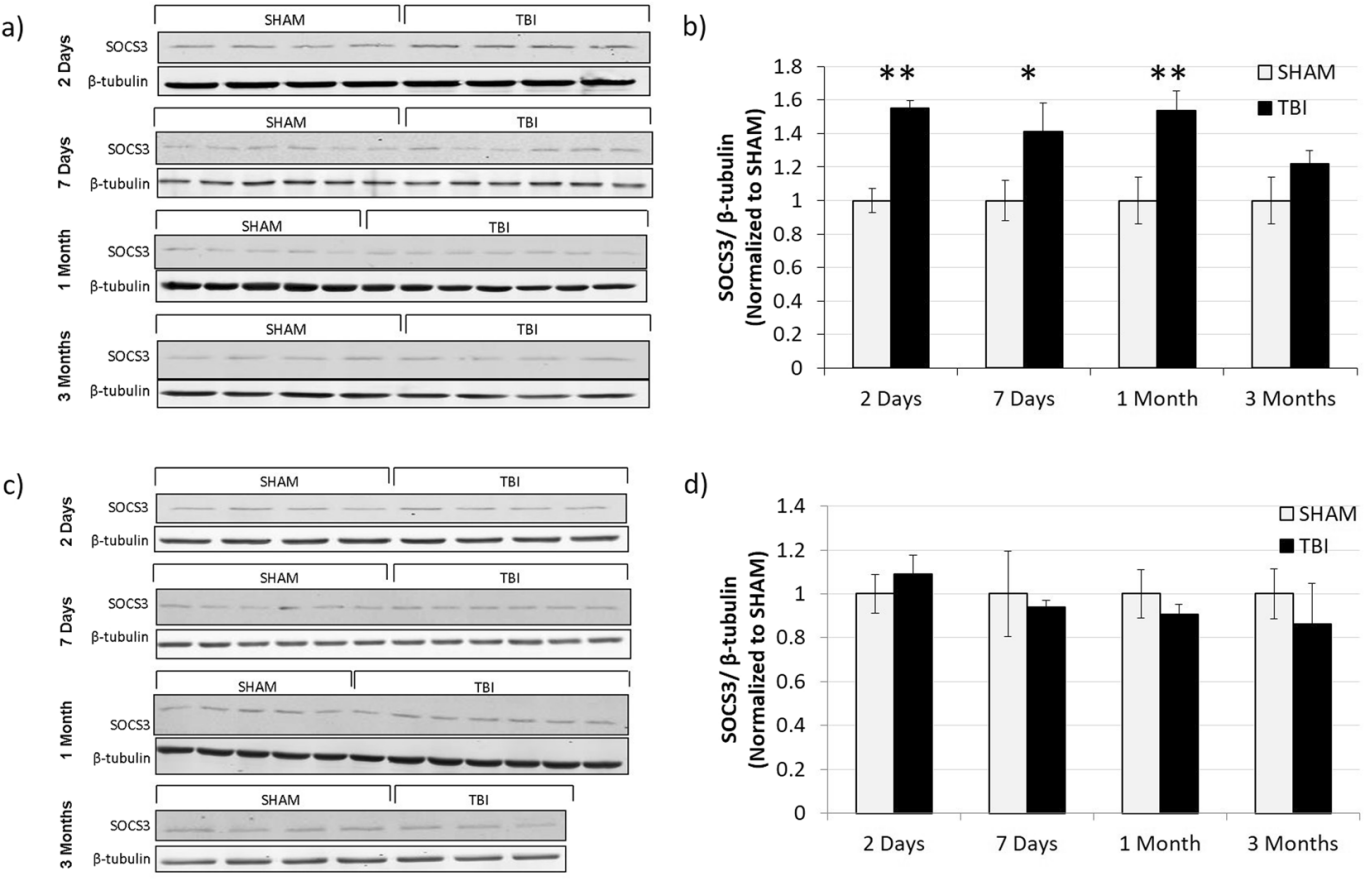
Transiently increased SOCS3 expression in the ipsilateral but not the contralateral hippocampus. (**a**) Western blots detecting SOCS3 in synaptosomes isolated from the ipsilateral hippocampus of 2 DPI (n = 4 for both sham and TBI), 7 DPI (n = 6 for both sham and TBI), 1 MPI (n = 5 sham, n = 7 TBI), and 3 MPI (n = 4 sham, n = 4 TBI) animals. **(b)** Quantitative graph of the Western blot analysis showing SOCS3 normalized to β-tubulin. Western blots **(c)** and corresponding quantification **(d)** from synaptosomes isolated from the contralateral hippocampus of 2 DPI (n = 4 for both sham and TBI), 7 DPI (n = 6 for both sham and TBI), 1 MPI (n = 5 sham, n = 7 TBI), and 3 MPI (n = 4 sham, n = 3 TBI) animals. Full length Western blots are presented in Supplementary Figs S5 and S6. Statistical significance was determined by unpaired t-test analysis. *p < 0.05; **p < 0.01.

To investigate whether the protein SOCS3 could be playing a role in the synaptic insulin resistance we saw in our TBI model, we determined the level of this protein in isolated synaptosomes from the ipsilateral (Fig. 8a,b) and contralateral (Fig. 8c,d) hippocampi at 2 DPI, 7 DPI, 1 MPI, and 3 MPI time points. In the ipsilateral hippocampus, we found a significantly increased level of SOCS3 at the synapse in TBI animals versus sham animals at 2 DPI (p = 0.0004), 7 DPI (p = 0.0411), and 1 MPI (p = 0.0079). In the contralateral hemisphere, however, we did not find altered levels of SOCS3 at the synapse at any of the time points.

## Discussion

TBI increases the risk of developing AD later in life^9,29^. Synaptic dysfunction caused by toxic Aβ and tau oligomers binding to the synapse and disrupting LTP properties is one of the initial events in AD leading to the cognitive decline that is associated with this disease^16,17^. For this reason, in the present work, we investigated alterations in the susceptibility of the synapse to Aβ and tau oligomer dysfunctional impact in an established rat model of TBI. Moreover, insulin signaling plays a role in synaptic health and function^30^, and its disruption through insulin resistance at the synapse has been shown to contribute to Aβ-induced spine loss in AD^18,31^. Thus, the main goals of the present study were (1) to determine whether synaptic insulin responsiveness after TBI is dysregulated and (2) to investigate related changes in synaptic vulnerability to the dysfunctional impact of Aβ and tau oligomers.

Using an *ex vivo* insulin stimulation method on isolated synaptosomes, we found the synaptic insulin receptor to have significantly decreased responsiveness as early as 7 days post-injury (DPI) after lateral FPI. This injury-promoted insulin resistance of hippocampal synapses became chronic as deficits were observed up to 3 months post-injury (MPI). While decreased insulin signaling in the CNS after mild TBI has previously been reported^28^, this is the first demonstration that insulin signaling is chronically impaired after a moderate TBI. Additionally, direct evaluation of the synaptic insulin receptor (IR), as performed here, bypasses assessment of the overall insulin signaling pathway and reveals that there is a chronic impairment at the level of the receptor.

In the ipsilateral hippocampus, we found a significant increase in synaptic IR levels at 3 MPI which could represent an attempted compensatory mechanism for the decreased insulin signaling here. However, these possible efforts were shown to be futile as we found that there was still a significantly decreased synaptic insulin response as well as decreased basal phosphorylation levels at this time point, indicating that there is a chronic alteration in the response of the receptor that cannot be overcome by upregulation of the receptor. On the other hand, we did not observe any changes in synaptic IR level at any of the time points for the contralateral hemisphere, possibly suggesting that any attempt to compensate for TBI-related synaptic insulin resistance is limited to the ipsilateral hemisphere.

In order to begin delving into the mechanisms responsible for driving the synaptic insulin resistance in TBI animals observed in our study, we determined levels of SOCS3. SOCS3 is a protein of the suppressor of cytokine signaling (SOCS) family that act as negative regulators of cytokine and growth factor signaling. We chose to look at SOCS3 due to its ability to be upregulated by inflammatory factors which have been found to be increased within hours after injury^32,33^. Specifically, SOCS3 protein expression can be induced by IL6 as well as IL10 and has been shown to negatively regulate insulin signaling^34^. Thus, SOCS3 was a good candidate to negatively regulate insulin signaling at early time-points, prior to the delayed (7 days) insulin resistance that we found. Furthermore, SOCS3 expression has been found to be upregulated in the CNS of AD patients^35^.

In the ipsilateral hippocampus, we found a significantly increased level of SOCS3 at the synapse in TBI animals versus sham animals at 2 DPI, 7 DPI, and 1 MPI as well as a trend toward increased levels at 3 MPI. Since increased levels of SOCS3 occur as early as 2 DPI, prior to the onset of synaptic insulin resistance that was observed starting only at 7 DPI, increased SOCS3 inhibition of the insulin receptor could potentially be a driving factor in initiating the insulin resistance ensuing at the synapses after TBI. Furthermore, SOCS3 upregulation could be one molecular link between the chronic inflammation seen after TBI^33,36^ and the insulin resistance that we have reported here in hippocampal synapses after injury.

In the contralateral hemisphere, however, we did not find altered levels of SOCS3 at the synapse at any of the time points studied here. Since we still found synaptic insulin resistance beginning at 7 DPI in this hemisphere too, it is possible that the two hemispheres suffer insulin resistance after TBI driven by different mechanisms, with SOCS3 playing a significant role only in the ipsilateral hemisphere. This hypothesis is supported by previous findings that the gene expression level of IL-6 signal transducer (IL6ST), a protein involved in the complex for IL-6 signaling, was increased at an acute time point in the ipsilateral hemisphere and decreased in the contralateral hemisphere after a lateral, moderate TBI^37^. Although further studies are needed to establish the exact mechanisms of insulin resistance induction, this scenario would be consistent with prior reports describing opposing bilateral changes in proteins involved in TBI-related secondary injuries after a unilateral TBI, specifically in pathways involved in cell death, survival, and inflammatory response^37,38^.

We next aimed to determine whether reduced synaptic insulin responsiveness in TBI animals was associated with altered synaptic vulnerability to extracellular Aβ and tau oligomers as well as oligomer-induced synaptic dysfunction. We used *ex vivo* binding methodologies to assess synaptic resistance/vulnerability to extracellular Aβ and tau oligomer binding. Our data indicates that hippocampal synapses are not more susceptible to Aβ oligomer binding at the chronic time points of 1 month or 3 months after moderate FPI. Similar results were obtained using tau oligomers, whereby synapses were not more susceptible to tau oligomer binding at the chronic time points of 1 month or 3 months after TBI in either hemisphere.

We further investigated synaptic vulnerability to oligomers by determining functional vulnerability through oligomer-induced LTP inhibition. Consistent with previous reports^39^, we found basal LTP levels to be chronically impaired in the side of injury after lateral, moderate FPI through 3 MPI. Our electrophysiological results are also consistent with our *ex vivo* binding experiments, suggesting that synaptic vulnerability to Aβ and tau oligomers is not increased after TBI as we did not find an increase in oligomer-induced LTP impairments compared to sham animals at 1 or 3 MPI.

Insulin treatment of brain slices concurrent with either Aβ or tau blocked the LTP impairment induced by the oligomers in hippocampal slices from both the ipsilateral and contralateral hemispheres from sham animals. This is consistent with the literature showing that insulin inhibits Aβ-induced impairment of LTP using the same concentrations, animal species, and electrophysiological set-up used here^40^. This group suggested that this beneficial effect of insulin was due to a direct interaction of Aβ and insulin whereby insulin inhibits the formation of soluble Aβ oligomers and thus prevents oligomer-induced LTP impairment in a manner that is independent of insulin receptor activation. While these two proteins may indeed interact to hinder Aβ oligomer formation, our results would argue against this conclusion since the beneficial effect of insulin that we observed in slices from sham animals was not seen against either Aβ or tau in ipsilateral hippocampal slices from TBI animals at both 1 and 3 MPI. Insulin treatment also did not block Aβ impairment at either time point in the contralateral hippocampus. We did find that insulin treatment partially blocked LTP reduction due to tau oligomers in the contralateral hippocampus at both chronic time points, yet we do not have an explanation for this phenomenon. This differential finding of insulin treatment in slices from sham and TBI animals suggests that activation of the insulin receptor does, in fact, contribute to the functional protection afforded by insulin against oligomer toxicity.

In conclusion, from our electrophysiology data as well as our *ex vivo* insulin stimulation analysis, we found chronically decreased insulin responsiveness of the synaptic insulin receptor in the hippocampus after moderate TBI that does not lead to increased synaptic susceptibility to Aβ or tau oligomers.

While there is consensus that intranasal insulin treatment benefits cognition by acting on neuronal insulin receptors to overcome resistance^41^, some have pointed out that indirect pathways may influence cognition, and, thus, neuronal insulin signaling may not be needed for cognitive enhancement via insulin therapy^41^. Evidence such as increased regional cerebral blood flow and enhanced cognition in the insulin-resistant type 2 diabetes patients^42^ supports this notion. In an opposing conclusion from animal models of type 2 diabetes where brain insulin resistance has been confirmed, insulin therapy acting on the hippocampus was found to be unsuccessful in improving cognition^43^. This could suggest that once brain insulin resistance has developed, insulin treatment may not be sufficient to overcome resistance at the cellular level^41^. Our results would support this overall conclusion in a TBI model showing that brain insulin resistance would negate the efficacy of insulin as a therapy to provide protection against oligomer-induced synaptic dysfunction in injured animals as well as in AD patients with a history of TBI.

## Methods

### Animals

Male Sprague-Dawley rats were utilized for all of the experiments in this study. All rats were 2–4 months old (300–450 grams) at the time of surgery/injury. All experimental protocols involving animals in this study were approved by Institutional Animal Care and Use Committee of the University of Texas Medical Branch, and all experiments were performed in accordance with relevant guidelines and regulations. Animals were housed under USDA standards (12:12 hour light dark cycle, food and water ad libitum) at the UTMB vivarium. After the designated amount of time after surgery/injury, the rats were sacrificed by isoflurane overdose and decapitated. The brains were quickly removed, dissected into major regions and opposing hemispheres (“ipsilateral” referring to the brain hemisphere that underwent the craniotomy in the lateral FPI procedure and “contralateral” for the opposite hemisphere), snap frozen, and stored at −80 °C until ready for further analysis.

### Parasagittal fluid-percussion injury

#### Craniotomy

Male Sprague-Dawley rats (300–450 grams) were anesthetized (4% isoflurane) and prepared for moderate or sham parasagittal fluid percussion injury (FPI). Rectal temperatures were monitored and maintained within a range of 37.5 ± 0.5 °C using an overhead lamp and a thermostatically controlled water blanket (Gaymar, Orchard Park, NY). Rats were placed in a stereotaxic apparatus, a midline incision of the skin was performed, and the skull was exposed. A craniotomy was performed using a 5 mm diameter Michele trephine at 1 mm lateral (right) to the sagittal suture, midway between the lambda and bregma. The bone chip was removed, leaving the dura intact. A modified 20-gauge needle hub was secured in place over the exposed dura with superglue and cemented into place with hygienic dental acrylic.

#### Parasagittal fluid percussion injury

TBI was administered by means of a fluid percussion injury device (AmScien Instruments, Richmond, VA). The animal’s craniotomy hub site was directly connected to the transducer end of the injury device. The pendulum of the device was lifted to a specified height to correspond to the intensity of injury desired, moderate injury level for these studies. The pendulum was released and struck the back of the sterile saline-filled cylinder causing a direct brain deformation injury. The fluid pressure pulse and the righting reflex time were recorded. At 2 days, 7 days, 1 month, or 3 months after TBI or sham injury, the rats were euthanized and brain tissue collected as described above.

The control sham animals used in this study underwent the craniotomy and connection to the fluid percussion injury device, just as the TBI animals, with the exception of the pendulum release.

### Synaptosomal isolation

Synaptosomes containing both pre- and post-synaptic components were isolated using SynPER (Thermo Scientific, Waltham, MA) with 1% protease and phosphatase cocktail inhibitors from frozen tissue that had been snap frozen on dry ice and transferred to −80 °C as described in Comerota, 2017^44^. Homogenate was centrifuged at 1,200 × g RCF for 10 minutes at 4 °C. Supernatant was collected and centrifuged at 15,000 × g RCF for 20 minutes at 4 °C.Synaptosomal pellets was resuspended in 48 μL HEPES-buffered Krebs-like (HBK) buffer (143-mM NaCl, 4.7-mM KCl, 1.3-mM MgSO_4_, 1.2-mM CaCl_2_, 20-mM HEPES, 0.1-mM NaH_2_PO_4_, and 10-mM D-glucose, pH 7.4). The quality of the synaptosomes are routinely verified by Western blot and electron microscopy as previously reported^45^.

### Insulin responsiveness

#### Insulin stimulation of synaptosomes

After isolation of synaptosomes, insulin stimulations were performed as previously described^45–47^. In short, 100 mM ATP stock was added for final concentration of 8 mM to synaptosomes in HBK, and U-100 insulin was added for 200 nM final insulin concentration. Samples were vortexed and incubated for 15 minutes at 37 °C. Synaptosomes were pelleted and resuspended in 1X RIPA (75-mM NaCl, 25- mM Na_2_PO_4_, 1-mM EDTA, 0.5% NP-40, and 0.5% TritonX-100) plus 1% protease and phosphatase cocktail inhibitors to solubilize the proteins for Western blot and Wes detection. Samples were then stored at −80 °C.

#### Wes analysis of insulin responsiveness

Wes is an automated, capillary-based electrophoresis system for the separation and analysis of proteins for quantitative data. Further information on the Wes instrumentation can be found https://www.proteinsimple.com/wes.html. Phosphorylation extent of the insulin receptors was analyzed using Wes (Protein Simple, San Jose, CA) with specific antibodies against the phosphorylated form of the 1150/1151 tyrosine residue of the insulin receptor (Cell Signaling Cat. #3024L). The phosphorylated form was normalized against β-tubulin (Cell Signaling Cat. #2146S). Another Wes was run for the total amount of IR (Cell Signaling Cat. #3025S) which was normalized to β-tubulin as well. The ratio of normalized phosphorylated-IR over normalized total IR was used to assess the extent of insulin responsiveness.

### Aβ-binding

#### Aβ oligomer preparation

Human Aβ_1–42_ peptide was purchased from Department of Biophysics and Biochemistry, Harvard University, MA, and Aβ oligomers were prepared from lyophilized synthetic Aβ aliquots as previously described^48^. Briefly, 200 µL of 1,1,1,3,3,3- Hexafluro-2-propanol (HFP) was used to dissolve the Aβ lyophilized aliquots of 0.3 mg. 700 µL of DDI water was then added, and a cap with four holes was placed on the tube. The sample was magnetically stirred under a fume hood for 48 hours. The Aβ oligomers were aliquoted, frozen at −80 °C, and used within 3 months. For the flow cytometry analysis of Aβ oligomer-binding to synaptosomes, Aβ oligomers spiked with Flour 647 tagged Aβ (AnaSpec Inc., Fremont, CA) were utilized. These Aβ oligomers were prepared by adding 7 µL of the tagged Aβ to the HFP-Aβ mixture, prior to oligomer formation. Thus, the tagged Aβ is distributed throughout the oligomers so that a single oligomer can be composed of both tagged and untagged Aβ. The quality of the oligomeric preparations was routinely checked by Western blot and dot blot analysis using 6E10 and A-11 antibodies (Aβ oligomer specific).

#### Aβ oligomer binding challenge

Oligomeric Aβ to be employed in the same experiment was always used from aliquots of the same batch of Aβ. Hippocampal synaptosomes were treated with Aβ oligomers for an *ex vivo* binding challenge and evaluated using flow cytometry. An equal number of isolated synaptosomes per animal determined by flow cytometry were pooled for each experimental group. For each group, the pooled samples were then aliquoted into 10 separate tubes containing 10 million synaptosomes each. This was repeated 3 times for 3 separate curves. Each sample was incubated with Aβ oligomers tagged with HyLite Fluor 647 for the desired μM concentration, ranging from 0 μM to 20 μM, for 1 hour at room temp. Synaptosomes were then pelleted, washed 3 times with HBK buffer, and resuspended in PBS. Data was acquired by a Guava EasyCyte flow cytometer (EMD Millipore, Burlington, MA) and analyzed using Incyte software (EMD Millipore).

### Tau-binding

#### Tau-oligomer preparation

Prepared recombinant tau oligomers were graciously given to us by Dr. Rakez Kayed’s laboratory. The tau oligomers were produced as previously described^49^.

#### *Ex vivo* tau-oligomer binding

Synaptosomes were treated with tau oligomers for an *ex vivo* binding challenge and evaluated using ELISA as previously performed in our lab^50^. Hippocampal synaptosomes from each animal were challenged and evaluated independently. Using flow cytometry, 10 million synaptosomes from each animal were aliquoted and challenged with 2 µM of tau oligomers for 1 hour at room temperature. The samples were then centrifuged and washed with HBK buffer 3 times to thoroughly remove any unbound tau oligomers. Synapse number was acquired using flow cytometry once again, and an equal amount of synaptosomes per sample were analyzed by tau5 ELISA.

#### ELISA analysis of tau

Total tau levels were measured by ELISA analysis using the total tau antibody, tau5 (Biolegend Cat. # 806401). Samples were incubated on the ELISA plate at 4 °C overnight. Samples were discarded and each well was washed with Tris-buffered saline with low Tween 20 (0.01%) (TBS-low T) followed by blocking with 10% nonfat milk for 2 hours. After a second wash, the primary tau5 antibody (1:1000 in 5% nonfat milk in TBS-low T) was incubated in each well for 1 hour at room temperature. After a third wash, the plates were incubated for 1 hour at room temperature with horseradish peroxidase-conjugated anti-rabbit IgG secondary antibody (1:10,000 in 5% nonfat milk in TBS-low T; Promega). Following the fourth wash, 3,3,5,5-tetramethylbenzidine (TMB-1 component substrate; Sigma-Aldrich) was added to each well. After 30 min of incubation in the dark, 1 M HCl was added to stop the reaction, and the plate was read at 450 nm for tau detection and quantification.

### Electrophysiology

Animals were euthanized with deep isoflurane anesthesia, decapitated by guillotine, and brains were harvested and sliced using Compresstome VF-300 (Precisionary Instruments, Greenville, NC) in NMDG-aCSF to obtain 450 μm transverse brain sections. Slices were allowed to recover for 10 minutes in NMDG-aCSF at 35 °C. Slices were then maintained at room temperature in a modified HEPES holding aCSF solution. Slices were recorded in standard recording aCSF. All solutions were aerated using 95% O_2_ with 5% CO_2_. For oligomer challenges, the slices were incubated for 1 hour at room temperature prior to recording with 200 nM Aβ oligomers, 50 nM tau oligomers, and/or 200 nM insulin. For slices treated with insulin, 200 nM insulin was also present in the aCSF recording solution. Evoked field excitatory post-synaptic potentials (fEPSPs) recordings were performed by stimulating the Schaffer collateral pathway using a stimulating electrode of 22 kΩ resistance placed in the CA3 region and recording in the CA1 region. LTP was induced using a high frequency stimulation protocol (3 × 100 Hz, 20 seconds) as previously described^51^. Recordings were digitized with Digidata 1550B (Molecular Devices, Sunnyvale, CA), collected using an Axon MultiClamp 700B differential amplifier (Molecular Devices), and analyzed using Clampex 10.6 software (Molecular Devices). Current stimulation was delivered through a digital stimulus isolation amplifier (A.M.P.I, ISRAEL) and set to elicit an fEPSP approximately 30% of maximum for synaptic potentiation experiments using platinum-iridium tipped concentric bipolar electrodes (FHC Inc., Bowdoin, ME). Baseline recordings were obtained by delivering single pulse stimulations at 20 second intervals. All data are represented as a percentage change from the initial average baseline fEPSP slope obtained for the 10 minutes prior to HFS.

### Western blot analysis for SOCS3

The bicinchoninic acid (BCA) assay method was used for protein estimation to prepare samples of equal protein concentration. Samples were prepared in 2-mercaptoethanol (2-ME) and boiled prior to loading. Thirty micrograms of protein were loaded with appropriate marker on 10% SDS-PAGE gels followed by transfer to Amersham Protran nitrocellulose transfer membrane (GE Healthcare-Life Sciences) for 1 hour at 100 V. The membrane was blocked using Odyssey blocking buffer (LI-COR, Lincoln, Nebraska) for 1 hour at room temperature. Primary antibodies were diluted 1:1000 in 1X TBST and incubated with the membrane at 4 °C overnight for SOCS3 (Cell Signaling Cat. #2923S) and 1 hour at room temperature for β-tubulin (Cell Signaling Cat. #2146S). The membrane was washed twice with 1X TBST for 15 minutes each and incubated with LI-COR secondary antibodies diluted at 1:10,000 in 1X TBST with 3% non-fat dry milk for 1 hour at room temperature. The membrane was again washed twice for 15 minutes each.

Western blots were imaged using LI-COR Odyssey infrared imaging system (LI-COR, Lincoln, Nebraska), application software version 3.0.30. The density of each immunoreactive band was measured using ImageJ software.

### Experimental design and statistical analysis

All data represented as mean ± SEM. All data were statistically analyzed using Graphpad Prism6. Unpaired t-tests were used to determine statistical significance between two groups for the insulin stimulations and oligomer binding experiments. Student’s t-tests were used in cases of equal variance and t-tests with Welch’s correction were used in cases of unequal variance. For the electrophysiology experiments, a one-way ANOVA with Bonferroni’s post hoc test was used to determine statistical significance between the LTP of each condition.

## Supplementary information

Supplementary Figures and Table

## Data Availability

The datasets used and generated in this study are available from the corresponding author on reasonable request.
