# Supplementary material for: Chronic synaptic insulin resistance after traumatic brain injury abolishes insulin protection from amyloid beta and tau oligomer-induced synaptic dysfunction
Source: Sci Rep. 2019 Jun 3;9:8228. doi: 10.1038/s41598-019-44635-z (PMC6546708; doi:10.1038/s41598-019-44635-z)
Supplement: Supplementary file 1 — Supplementary Figures and Table [file 41598_2019_44635_MOESM1_ESM.pdf]

Supplementary Information

For

Chronic synaptic insulin resistance after traumatic brain injury abolishes insulin protection from amyloid beta and tau oligomer-induced synaptic dysfunction

Whitney Franklin, Balaji Krishnan, Giulio Taglialatela

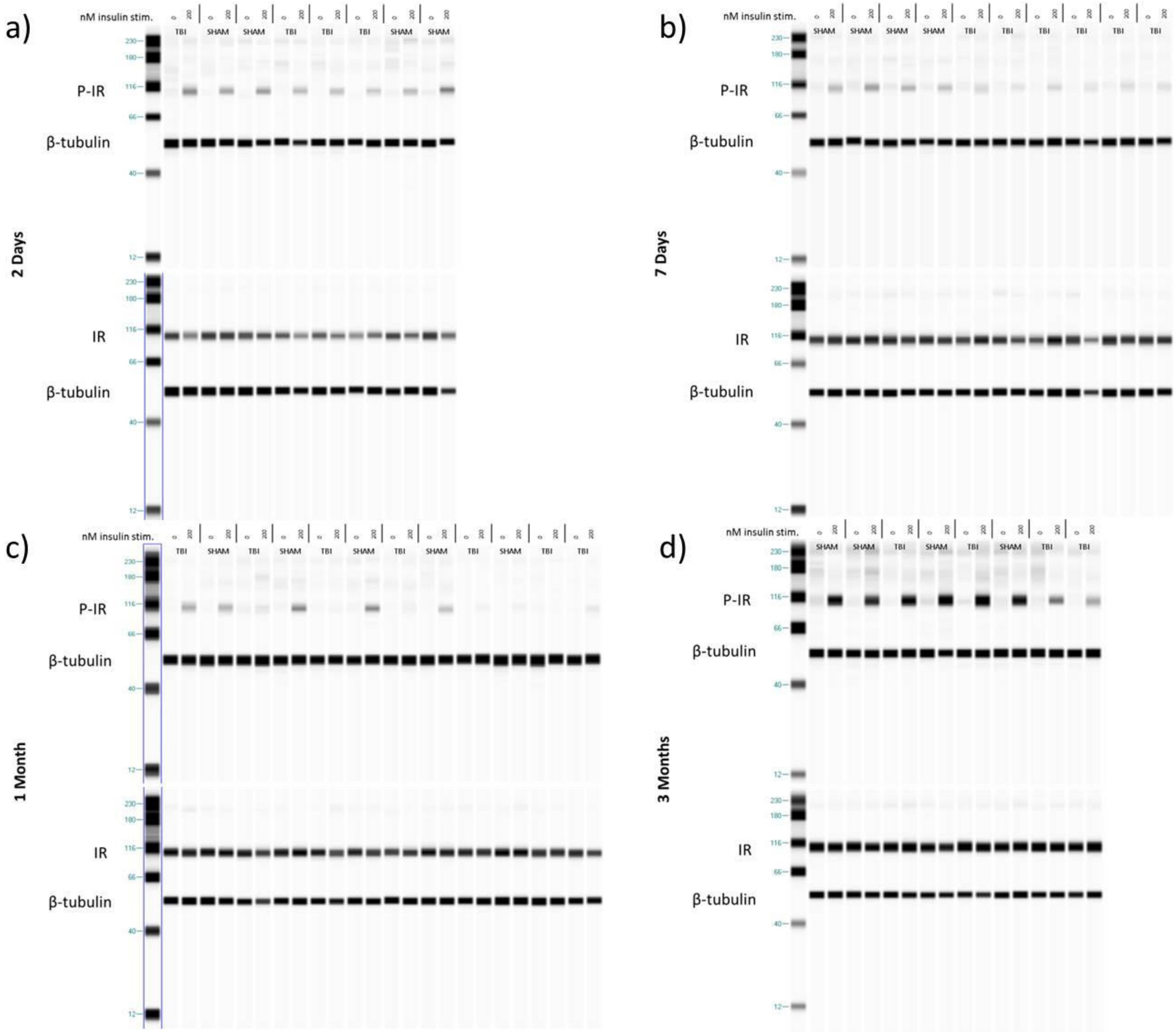

Supplementary Figure S1: Uncropped WES analysis of Insulin Responsiveness in Ipsilateral Hippocampi (Figure 1a). WES analysis of insulin-stimulated and unstimulated synaptosomes isolated from the ipsilateral hippocampus at **(a)** 2 DPI (n =4 for both sham and TBI), **(b)** 7 DPI (n= 4 sham, n=6 TBI), **(c)** 1 MPI (n= 5 sham, n=7 TBI), and **(d)** 3 MPI (n= 4 for both sham and TBI) animals. β-tubulin was used as a loading control for each WES run.

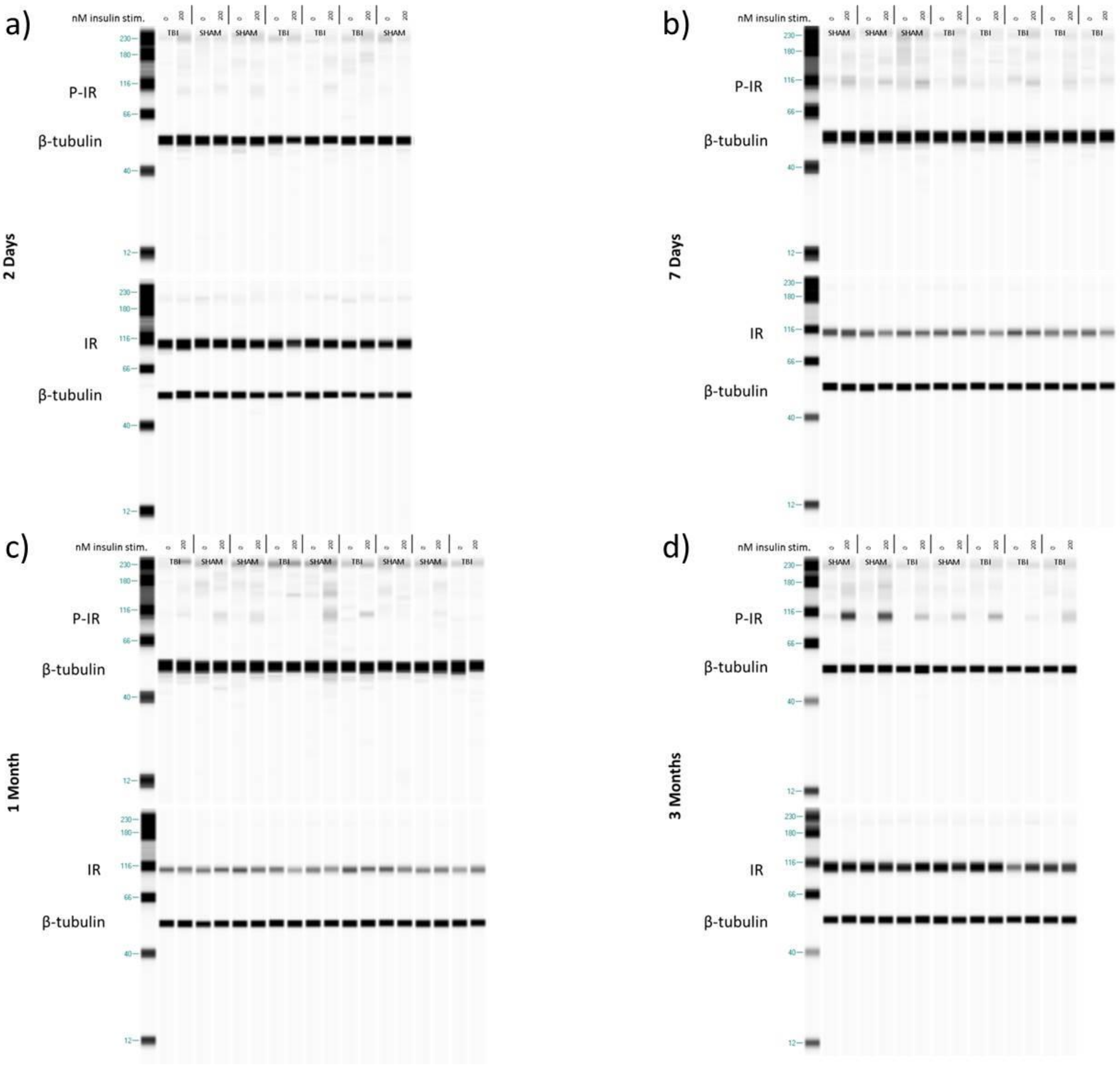

Supplementary Figure S2: Uncropped WES analysis of Insulin Responsiveness in Contralateral Hippocampi (Figure 1e). WES analysis of insulin-stimulated and unstimulated synaptosomes isolated from the ipsilateral hippocampus at **(a)** 2 DPI (n =3 sham, n=4 TBI), **(b)** 7 DPI (n= 3 sham, n=5 TBI), **(c)** 1 MPI (n= 5 sham, n=4 TBI), and **(d)** 3 MPI (n= 3 sham, n= 4 TBI) animals. β-tubulin was used as a loading control for each WES run.

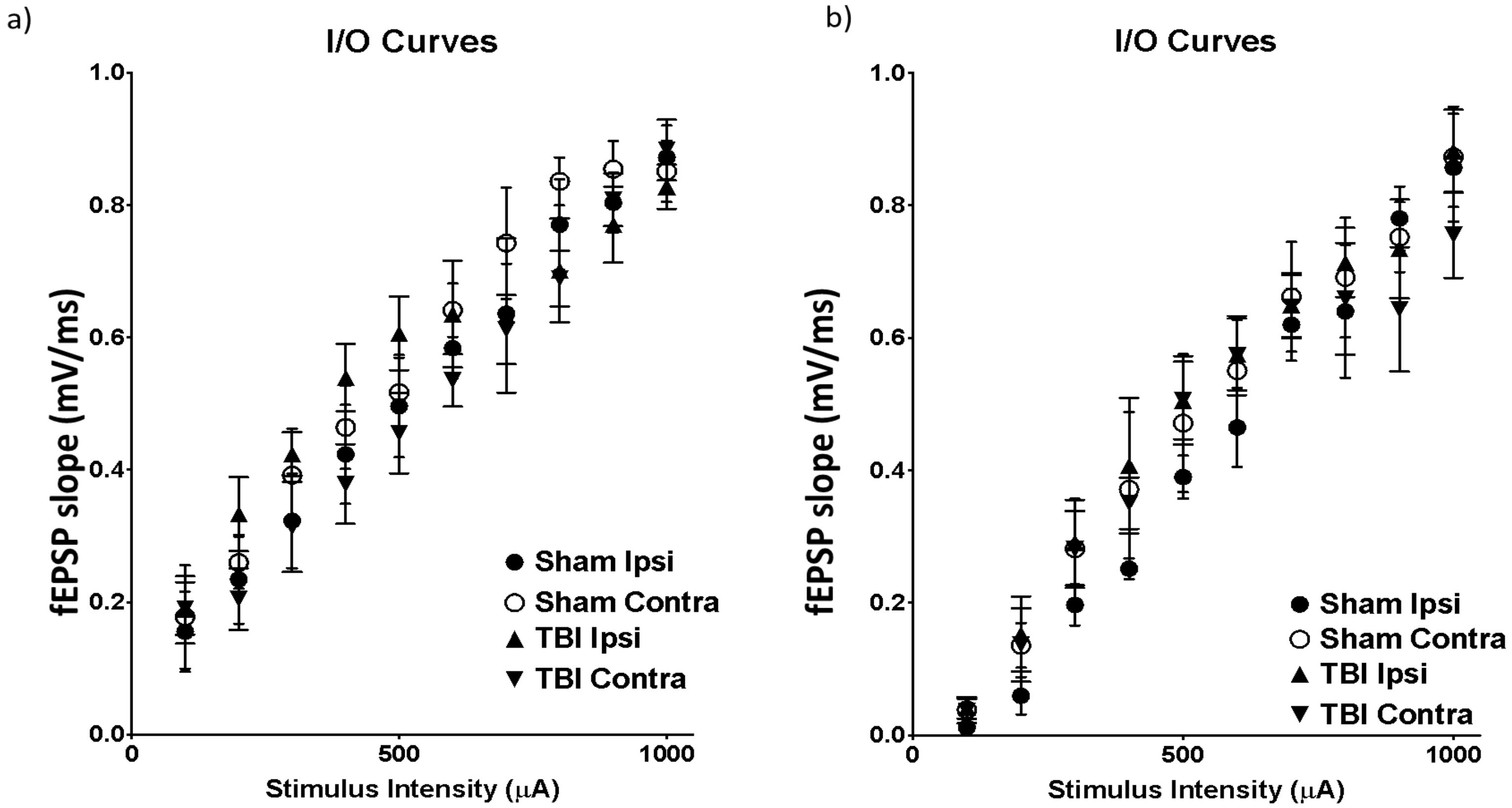

Supplementary Figure S3: Input/output curves for ipsilateral and contralateral hippocampi for sham and TBI animal slices. The fEPSP slope (mV/ms) obtained at increasing stimulus intensities (μA) at both **(a)** 1 month post-injury and **(b)** 3 months post-injury show no significant differences in the basal synaptic strength. 1 MPI n= 4 animals; 3 MPI n= 3-5 animals. Two-way ANOVA with Tukey's post hoc analysis was used to determine statistical significance. [For 1 MPI: $F_{3, 12}$=0.4943 P=0.6929] [For 3 MPI: $F_{3, 15}$=0.3794 P=0.7692]. Error bars represent standard error.

	Stimulus Intensity (µA)	Sham Ipsi N=4		Sham Contra N=4		TBI Ipsi N=4		TBI Contra N=4	
		Mean	SEM	Mean	SEM	Mean	SEM	Mean	SEM
1 Month Post-Injury	100	0.15538	0.06061	0.17774	0.07839	0.18857	0.05088	0.19052	0.03968
	200	0.23462	0.06699	0.25993	0.03961	0.33334	0.056	0.2046	0.04662
	300	0.32319	0.0717	0.39207	0.07035	0.424	0.03303	0.3137	0.06839
	400	0.4236	0.07473	0.4642	0.06285	0.53959	0.05103	0.3788	0.06026
	500	0.4963	0.0773	0.51687	0.05246	0.60612	0.05584	0.45581	0.06096
	600	0.58383	0.04578	0.64143	0.04051	0.63561	0.08049	0.53544	0.03988
	700	0.63621	0.0753	0.74263	0.08424	0.63347	0.11664	0.61223	0.05223
	800	0.77062	0.06893	0.83599	0.03643	0.70151	0.07803	0.6889	0.04207
	900	0.80377	0.0453	0.85449	0.0425	0.77073	0.05749	0.80816	0.04009
	1000	0.87211	0.04834	0.85124	0.04608	0.82797	0.03364	0.88349	0.04598

	Stimulus Intensity (µA)	Sham Ipsi N=3		Sham Contra N=3		TBI Ipsi N=5		TBI Contra N=8	
		Mean	SEM	Mean	SEM	Mean	SEM	Mean	SEM
3 Months Post-Injury	100	0.01205	0.00576	0.03876	0.01862	0.04144	0.01613	0.03684	0.01841
	200	0.05983	0.02806	0.13585	0.03354	0.15318	0.05669	0.13649	0.05556
	300	0.19674	0.03101	0.28162	0.07565	0.29104	0.06446	0.28094	0.05795
	400	0.25149	0.01553	0.37158	0.11674	0.40737	0.10237	0.35058	0.03902
	500	0.38991	0.03247	0.47136	0.10418	0.50553	0.05869	0.50577	0.06681
	600	0.46487	0.05972	0.55063	0.07699	0.57517	0.05408	0.57327	0.05971
	700	0.62002	0.05415	0.66217	0.08278	0.6497	0.04848	0.64719	0.04798
	800	0.64021	0.10051	0.69131	0.09032	0.71411	0.05218	0.65916	0.08442
	900	0.78051	0.04833	0.75233	0.053	0.73439	0.0745	0.64343	0.09388
	1000	0.85716	0.08151	0.8735	0.07562	0.88217	0.06257	0.75534	0.06505

Supplementary Table S1: Input-Output Averages for ipsilateral and contralateral hippocampi for sham and TBI animal slices. The averages of the slopes (mV/ms) measured after increasing stimulus intensities. There was no change in the slopes. N= number of slices per condition. Two-way ANOVA with Tukey's post hoc analysis was used to determine statistical significance. [For 1 MPI: $F_{3, 12}$=0.4943 P=0.6929] [For 3 MPI: $F_{3, 15}$=0.3794 P=0.7692].

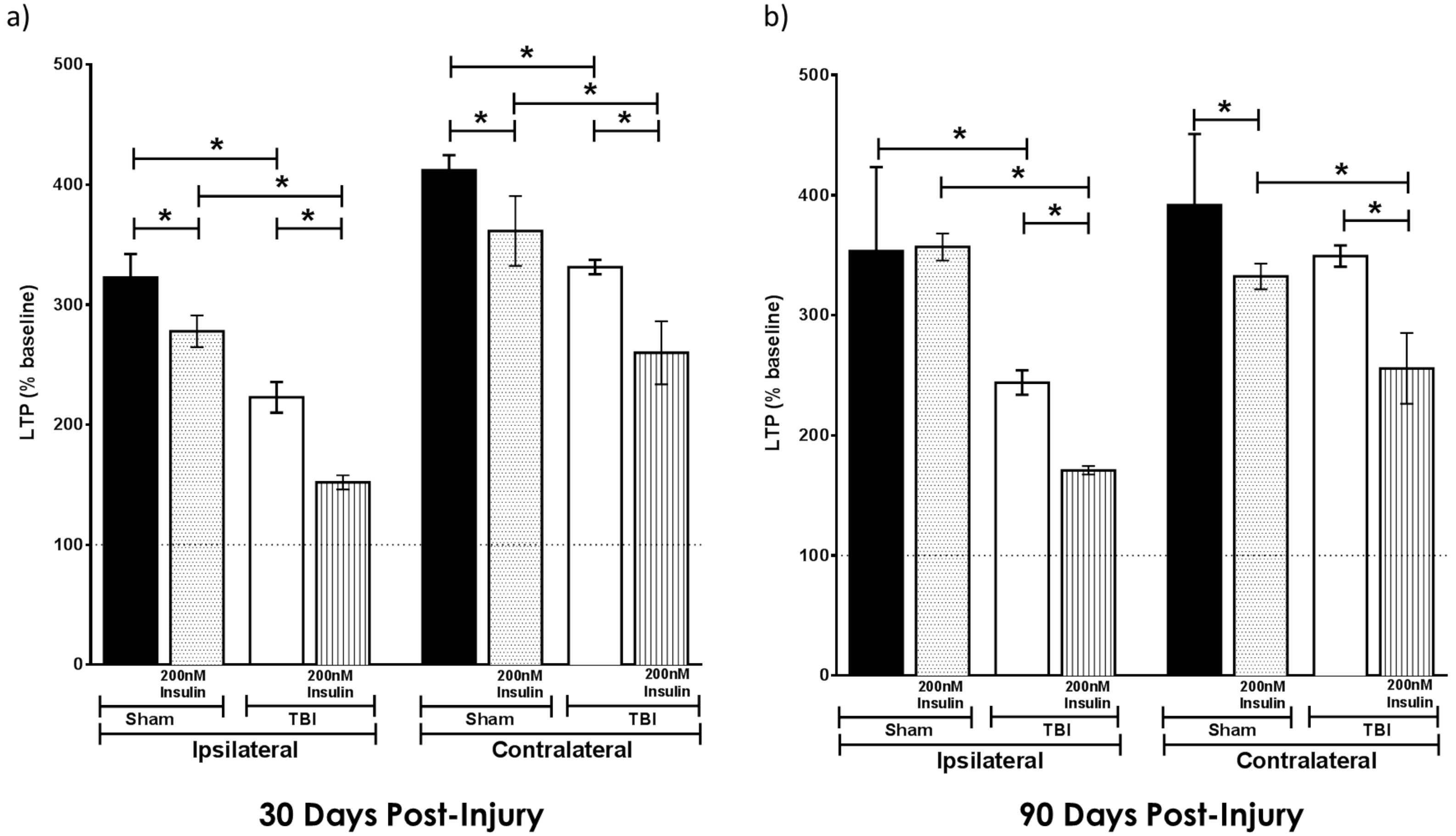

Supplementary Figure S4: Long-term potentiation (LTP) in hippocampal slices either untreated or exposed to an acute insulin treatment. Schaffer collateral field recordings were performed to measure LTP in slices from sham and TBI animals. Graphs showing the average of the fEPSP slope for the final 10 minutes (time points 60-70 minutes post high frequency stimulation) as an indication of LTP for each condition at **(a)** 1-month post-injury and **(b)** 3 months post-injury. 1 MPI n= 4 animals and 3-6 slices per condition; 3 MPI n= 3-5 animals and 3-7 slices per condition. One-way ANOVA with Bonferroni's post hoc analysis was used to determine statistical significance. Error bars represent standard error. *$p < 0.05$.

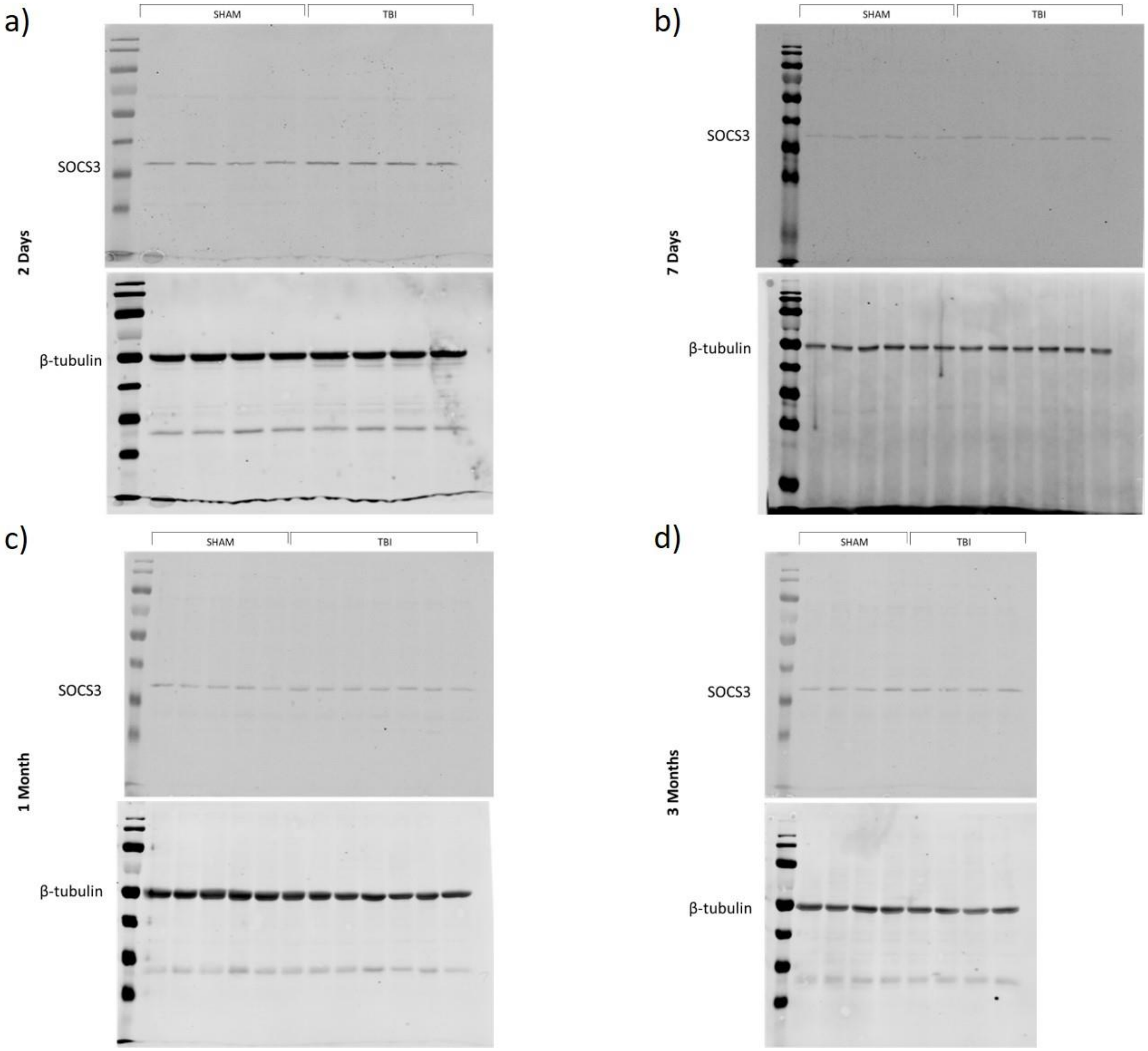

Supplementary Figure S5: Uncropped Western Blot Analysis of Synaptosomes From Ipsilateral Hippocampi (Figure 8a). Western blots probed with SOCS3 antibody and reprobed using β-tubulin antibody as a total loading control for each sample from the ipsilateral hippocampus at **(a)** 2 DPI (n =4 for both sham and TBI), **(b)** 7 DPI (n =6 for both sham and TBI), **(c)** 1 MPI (n= 5 sham, n=7 TBI), and **(d)** 3 MPI (n= 4 for both sham and TBI) animals.

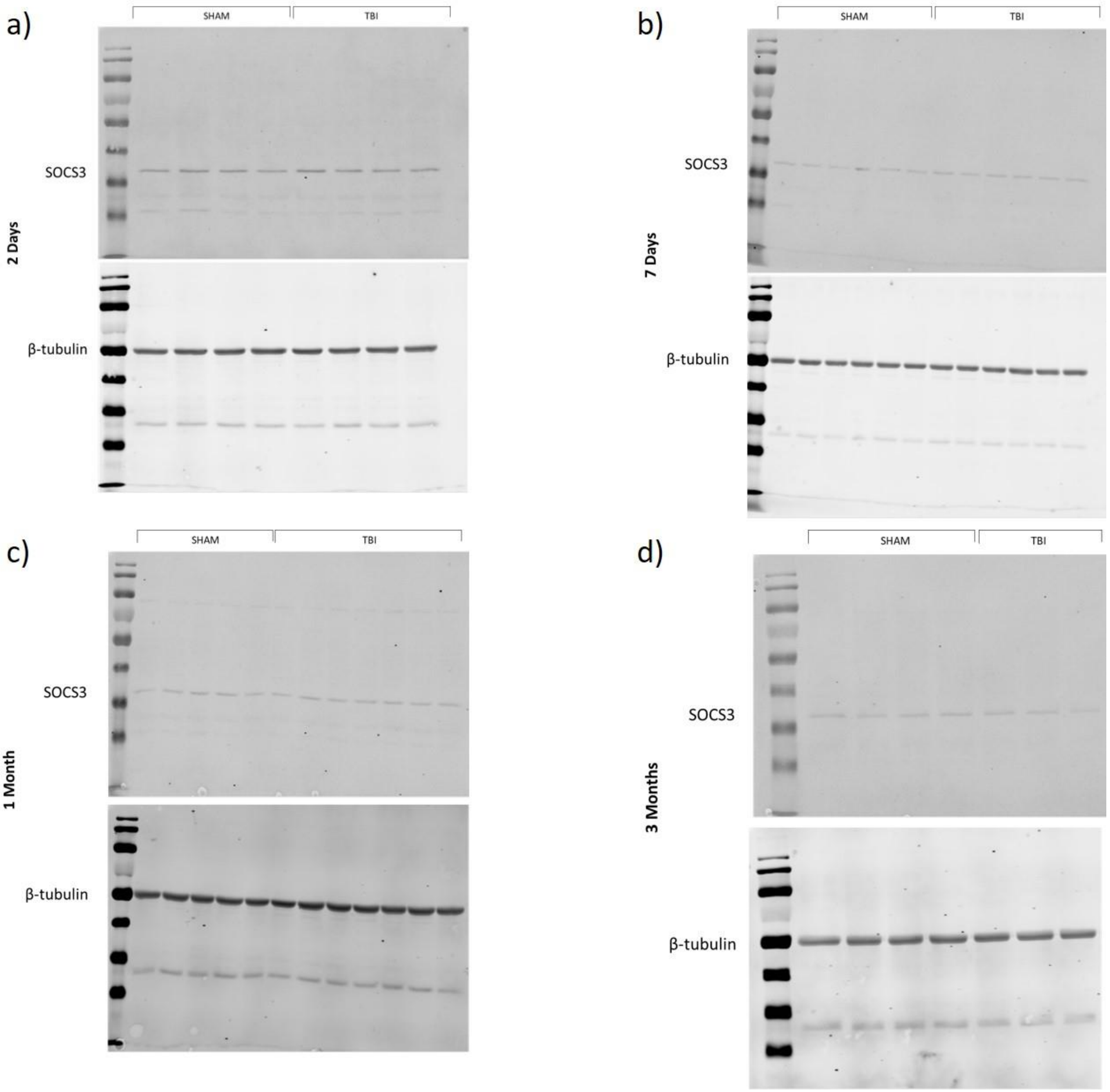

Supplementary Figure S6: Uncropped Western Blot Analysis of Synaptosomes From Contralateral Hippocampi (Figure 8c). Western blots probed with SOCS3 antibody and reprobed using β-tubulin antibody as a total loading control for each sample from the contralateral hippocampus at **(a)** 2 DPI (n =4 for both sham and TBI), **(b)** 7 DPI (n =6 for both sham and TBI), **(c)** 1 MPI (n= 5 sham, n=7 TBI), and **(d)** 3 MPI (n= 4 sham, n=3 TBI) animals.
